# Metal-Oxide-Semiconductor Nanostructure/NiO Microparticle Heterojunctions Formed on Metallic Foils for Sensitive Chemiresistive Ion Sensors

**DOI:** 10.1021/acsomega.5c02828

**Published:** 2025-06-20

**Authors:** Yoshinari Kimura, Hironori Tohmyoh

**Affiliations:** Department of Finemechanics, Graduate School of Engineering, Tohoku University, 6-6-01 Aoba, Aramaki, Aoba-ku, Sendai 980-8579, Japan

## Abstract

Chemiresistive metal-oxide-semiconductor (MOS) sensors for routine health and environmental monitoring are required to detect ion species in solutions simply and highly sensitively. In this study, *p*–*p* and *p*–*n* heterojunctions, formed by using facile processing, were introduced to enhance the detection sensitivity of MOS-based ion sensors. NiO microparticle-bearing CuO*_*x*_*-, ZnO-, and SnO*_*x*_*-based ion sensors were thermally and hydrothermally fabricated on metallic foils. On the MOS nanostructures, NiO microparticles affected the sensitivity of chloride, sodium, and calcium ion detection in solutions. The ZnO sensor with high-number density NiO microparticles demonstrated an excellent detection sensitivity of 639 at 30 °C in a 1 ppm chloride ion solution. These results suggest that bulk and surface reactions between the MOS and ionic solutions were enhanced by *p*–*p* and *p*–*n* heterojunctions, lowering activation energies. MOS/NiO *p*–*p* and *p*–*n* heterojunctions fabricated using the proposed process on metallic foils could contribute to the practical application of chemiresistive MOS sensors for detecting ionic solutions.

## Graphical abstract

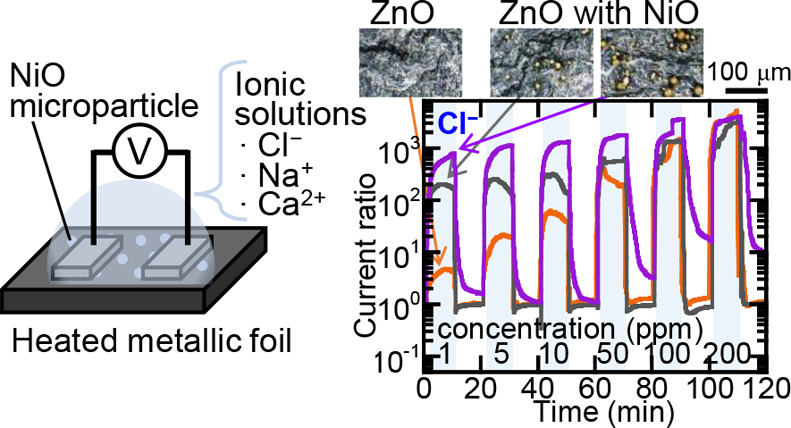

## Introduction

The detection of ionic species in human body fluids and industrial wastewater is necessary in various fields, including medicine and industry.^1–3^ Highly sensitive and accurate techniques for detecting ions in solutions, such as liquid chromatography,^4^ surface-enhanced Raman spectroscopy,^5^ and laser-induced breakdown spectroscopy,^6^ have been reported. There is also research on compact techniques for detecting ions, such as colorimetric^7^ and supramolecular organic frameworks.^8^ However, these techniques are limited in their applications and the situations in which they can be used because they require expensive equipment, complex measurement procedures, and advanced operational skills. In contrast, because of their easy application for measurements and miniaturization capability, ion-sensitive field-effect transistor,^9^ electrochemical,^10^ and chemiresistive^11,12^ type sensors have attracted considerable attention as electrical ion sensors. Among them, chemiresistive sensors are simply fabricated and detect ions in traces of solutions and, therefore, are suitable for low-cost ion detection for routine health and environmental monitoring applications.

Metal-oxide-semiconductor (MOS) materials are widely used as chemiresistive sensors, because of their low cost, ease of integration, and excellent thermal/chemical stabilities.^13,14^ Among MOS materials, CuO, ZnO, and SnO_2_ show great potential for the detection of various gases, including toxic and organic molecules.^15,16^ Because of the enhanced reaction between MOS surfaces and target molecules at high operating temperatures, MOS-based sensors can highly sensitively and rapidly detect molecules.^17,18^ This is a critical problem for detecting ionic solutions. MOS surface modifications, including nanostructure,^19–21^ heterojunction,^22–24^ and elemental doping,^25,26^ are effective methods for enhancing the sensitivity of MOS-based sensors at room temperature. For example, at room temperature, CuO nanoplatelets,^21^ ZnO/CuO *p*–*n* heterojunctions,^22^ and Fe-doped ZnO nanoparticles^25^ highly sensitively detect NO_2_, NH_3_, and HCHO gases, respectively. However, these MOS surface modification processes must be simplified and economized for low-cost ion detection. In our previous studies, we reported that MOS nanostructures and SnO*_*x*_*/NiO heterojunctions, formed using facile thermal and then hydrothermal processing on metallic foils, effectively detected ionic solutions at room temperature.^27,28^ A detailed understanding and further enhancement of the reaction between the MOS and ionic solutions could contribute to the practical application of MOS-based chemiresistive ion sensors.

In this study, we investigated the sensing performance of ion sensors fabricated using MOS nanostructure/NiO microparticle *p*–*p* or *p*–*n* heterojunctions formed using facile thermal and hydrothermal processing on metallic foils. The ion sensors demonstrated current changes in different concentrations of ionic solutions at 10–50 °C. According to these sensing performances, the bulk and surface reactions between MOS and ionic solutions were discussed.

## Experimental Section

### Formation of MOS/NiO Heterojunctions on Metallic Foils

NiO microparticle-bearing CuO*_*x*_*, ZnO, and SnO*_*x*_* nanostructures were thermally and hydrothermally formed on metallic foils. Figure 1a shows the formation process of the MOS/NiO heterojunctions. Cu (99.96%, 0.1 mm thick), Zn (99.2%, 0.3 mm thick), and Sn (99.9%, 0.3 mm thick) foils (The Nilaco Corporation) were used as substrates. First, those metallic foils were cut into 7 × 7 mm^2^ squares using scissors and then ultrasonically cleaned using deionized (DI) water for 10 min. To form the MOS nanostructures, the metallic foils were heated at 10 °C min^–1^ to 600 °C in air in an electric furnace and held there for 5 h. Next, colloidal solutions were prepared by mixing either 1 or 4 mg of Ni­(OH)_2_ (95.0%, FUJIFILM Wako Pure Chemical) with 2 mL of ethanol. Finally, the NiO microparticles were deposited by drop-casting 0.1 mL of the colloidal solution on the MOS nanostructures’ surfaces with a micropipet and then immediately heating in air in an electric furnace at 20 °C min^–1^ to 600 °C and held there for 5 h. This hydrothermal process was also used to prepare NiO microparticle-free samples. The NiO microparticle-free and -bearing nanostructured samples were named as OX and Y-OX/NiO, respectively, where OX is the MOS material (CuO*_*x*_*, ZnO, or SnO*_*x*_*), and Y is the Ni­(OH)_2_ weight.

**Figure 1:**
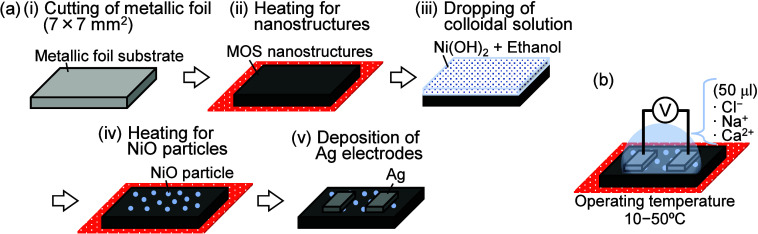
Schematics of (a) the fabrication process of NiO microparticle-bearing ion sensors and (b) the measurement setup of the ion sensors.

### Material Characterization

The morphologies of the MOS nanostructures and NiO microparticles were characterized by using field-emission scanning electron microscopy (SEM; SU-70, Hitachi High-Tech) and optical microscopy (RX-100, Hirox). The elemental compositions of the sample surfaces were analyzed by using energy-dispersive X-ray spectroscopy (EDX; Aztec Energy X-max, Oxford Instruments) and X-ray diffraction (XRD; SmartLab, Rigaku) with a Cu Kα radiation source.

### Fabrication and Evaluation of Ion Sensors

Ion sensors were fabricated by depositing two 2 mm wide Ag electrodes at an interelectrode gap of 0.5 mm on MOS nanostructures using conductive paste. The sensors were evaluated using 1–200 ppm chloride (Cl^–^), sodium (Na^+^), and calcium (Ca^2+^) ionic solutions at various operating temperatures, as shown in Figure 1b. The concentration of the ionic solution was adjusted using the ion standard solution (FUJIFILM Wako Pure Chemical) and DI water (resistivity of 8 MΩ cm or more). Ion sensing performance was investigated using a source meter (model 2450, Keithley) to record the transient response of the electrical characteristics at an applied bias of 0.1 V. The electrical characteristics were measured while using a micropipet to drop 50 μL of the ionic solution onto the sensors’ surfaces and then using a blower to remove the solution droplet. The sensors’ detection sensitivities were calculated as *I*_Ion_/*I*_Air_, where *I*_Ion_ and *I*_Air_ are the currents measured with and without the ionic solution droplet on the sensors’ surfaces, respectively. The response time was defined as the length of time required to change the current by 90% after dropping the ionic solution on the sensors’ surfaces.

## Results and Discussion

### Material Characterization

The MOS nanostructures and NiO microparticles thermally and hydrothermally formed on the metallic foils’ surfaces were observed. Figure 2a–d shows SEM images of the morphologies of the CuO*_*x*_*, 4-CuO*_*x*_*/NiO, 4-ZnO/NiO, and 4-SnO*_*x*_*/NiO sample surfaces. As shown in Figure 2a,b, the heated Cu foil surfaces were covered with numerous large nanowires. In addition to the nanowires, microparticles with diameters of 11.9 ± 4.6 μm were observed on the 4-CuO*_*x*_*/NiO sample surface, as shown in Figure 2b. Small nanowires and nanoparticles were formed on the heated Zn and Sn foil surfaces (Figure 2c,d), respectively. As on the surfaces of the hydrothermally treated Cu foils, microparticles were also formed on the surfaces of the hydrothermally treated Zn and Sn foils. Figure S1 shows the SEM images of the morphologies of all of the sample surfaces. In Figure 2e, the microparticle number density is plotted as a function of the Ni­(OH)_2_ weight, and the microparticles were counted in the microscopy images in the inset of Figures 2e and S1. With an increasing Ni­(OH)_2_ weight, although the microparticle number density increased to approximately 470 mm^–2^ on the heated metallic foil surfaces, the microparticle diameter did not change. Thus, material-dependent nanostructures and number density-controlled microparticles were thermally and hydrothermally formed on the metallic foil surfaces, respectively.

**Figure 2:**
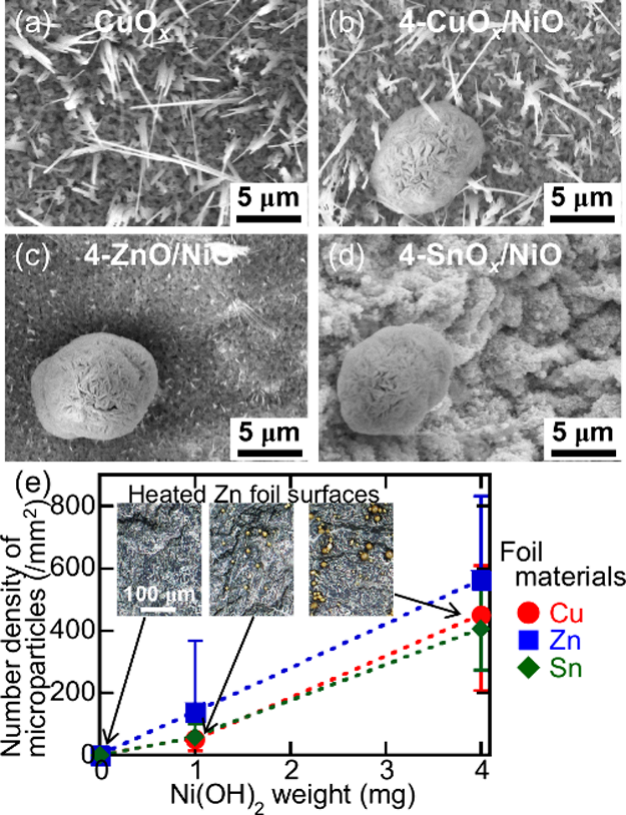
SEM images of the (a) CuO_*x*_, (b) 4-CuO_*x*_/NiO, (c) 4-ZnO/NiO, and (d) 4-SnO_*x*_/NiO sample surfaces. (e) The number density of the microparticles deposited on the heated metallic foil surfaces as functions of the Ni­(OH)_2_ weight. The insets show the corresponding optical microscopy images of the heated Zn foil surfaces.

Figure 3a–c shows the EDX spectra of the nanostructures and microparticles formed on the 4-CuO*_*x*_*/NiO, 4-ZnO/NiO, and 4-SnO*_*x*_*/NiO sample surfaces. In addition to Cu, Zn, and Sn atoms, O atoms were detected in the nanostructured regions on the heated Cu, Zn, and Sn foil surfaces, respectively. The corresponding atomic ratios were 50.3:49.7, 55.1:44.9, and 30.4:69.6 for Cu:O, Zn:O, and Sn:O. On all the metallic foil surfaces, the nanostructures were sufficiently oxidized. Figure 3d shows the XRD patterns of the CuO*_*x*_*, ZnO, and SnO*_*x*_* samples. The XRD patterns exhibited few Cu, Zn, and Sn peaks. The heated Cu, Zn, and Sn foil surfaces predominantly comprised CuO and Cu_2_O, ZnO, and SnO_2_ and SnO, respectively. The Ti and Ti_2_O_3_ peaks observed in the ZnO-based samples originate from the elements of the equipment. On the other hand, the microparticle regions of all the samples contained Ni and O atoms, as shown in Figure 3a–c, and regardless of the metallic foil material and Ni­(OH)_2_ weight, the Ni:O atomic ratio was always 50.4:49.6. Furthermore, compared to the XRD pattern of the ZnO sample, that of the 4-ZnO/NiO sample exhibited NiO peaks (Figure 3e). Thus, the microparticles hydrothermally deposited on the heated metallic foil surfaces comprised NiO. The EDX spectra and XRD patterns of other samples are shown in Figure S2. These results suggest that thermal and then hydrothermal processes on metallic foils are effective in forming MOS nanostructures and controlling the NiO microparticle number density.

**Figure 3:**
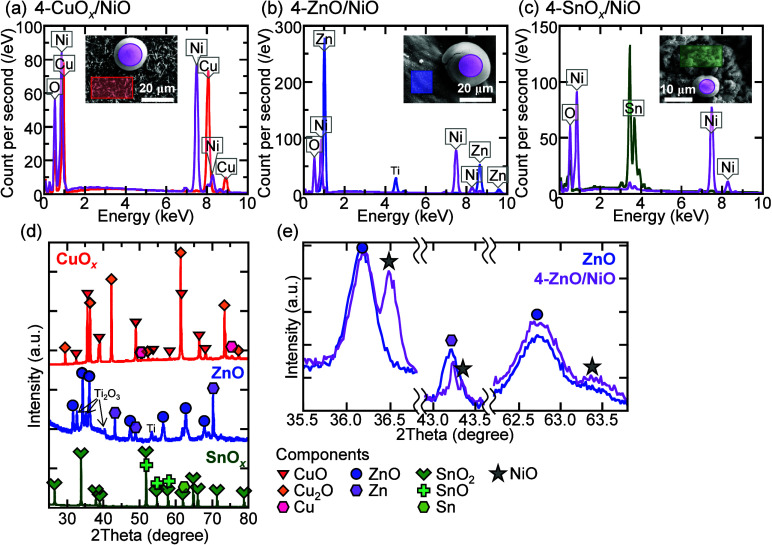
EDX spectra of the (a) 4-CuO_*x*_/NiO, (b) 4-ZnO/NiO, and (c) 4-SnO_*x*_/NiO samples and the corresponding SEM images are shown in the insets. The XRD patterns of (d) CuO_*x*_, ZnO, and SnO_*x*_ and (e) ZnO and 4-ZnO/NiO samples.

### Ion Sensing Performance

The ion sensing performance of the sensors fabricated using different MOS materials and NiO microparticle number densities was evaluated by using different concentrations of Cl^–^, Na^+^, and Ca^2+^ solutions. Figure 4 shows the transient electrical responses of the sensors to 1–200 ppm of Cl^–^ and Na^+^ solutions at 30 °C. To compare the transient electrical responses of the different sensors, the current ratio *I*_Meas_/*I*_Air_init_ was used, where *I*_Meas_ is the current measured in the solution and air, and *I*_Air_init_ is the initial current measured in air. For all the sensors, the current changed and then recovered to its initial value after dropping the ionic solution on and then removing it from the sensors’ surfaces, respectively. For all the ionic solutions, the sensors’ currents increased with increasing ion concentration. After dropping to 1 ppm of Cl^–^ and Na^+^ solutions, the CuO*_*x*_* and 1-CuO*_*x*_*/NiO sensors’ currents decreased, and the 4-CuO*_*x*_*/NiO sensor’s currents increased from the currents measured in air. The ZnO-based sensors possessed unique properties with respect to the NiO microparticle number density and ionic solution concentration. With increasing Cl^–^ concentration, the current ratios of the ZnO, 1-ZnO/NiO, and 4-ZnO/NiO sensors in the solutions increased from 4.3 to 3700, 170 to 3000, and 650 to 4000, respectively. The high-number density NiO microparticles effectively detected 1 ppm of Cl^–^ solutions. In contrast, the ZnO sensor possessed the highest current ratio for the 1 ppm of Na^+^ solution. The NiO microparticles did not affect the ZnO-based sensors’ currents in highly concentrated ionic solutions. The SnO*_*x*_*-based sensors’ currents in 1 and 200 ppm ionic solutions were lower and higher than those in air, respectively. The NiO microparticle-bearing SnO*_*x*_* nanostructures enhanced the current ratios of the sensors in the ionic solutions. For all of the sensors, the transient electrical responses measured in Ca^2+^ (Figure S3) showed the same trend as in Na^+^.

**Figure 4:**
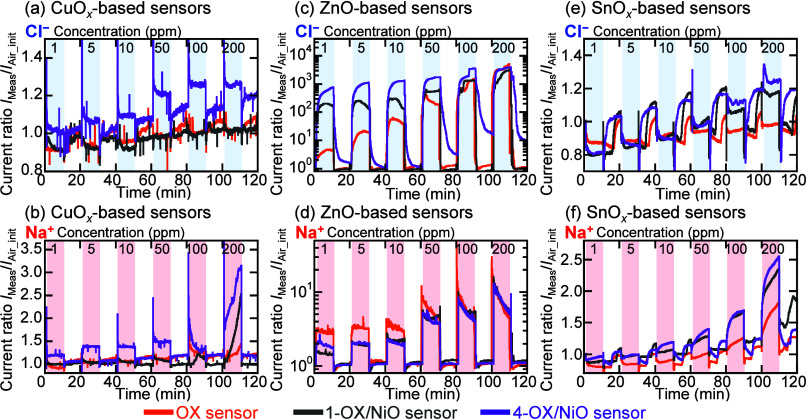
Transient electrical responses of the (a, b) CuO_*x*_-, (c, d) ZnO-, and (e, f) SnO_*x*_-based sensors at different Cl^–^ and Na^+^ concentrations, respectively.

Figure 5 shows the sensors’ detection sensitivities and response times plotted as functions of Cl^–^ and Na^+^ concentrations. The detection parameters of all of the sensors in Ca^2+^ solutions are shown in Figure S3. With increasing concentrations of all of the ionic solutions, the detection sensitivity of all of the sensors increased (Figures 5a–c and S3). The detection sensitivities of the 4-CuO*_*x*_*/NiO sensor in 200 ppm of Cl^–^, Na^+^, and Ca^2+^ solutions were 1.1, 2.5, and 4.6, respectively. The CuO*_*x*_*-based sensors with higher-number-density NiO microparticle possessed higher detection sensitivities. The detection sensitivities of the 4-ZnO/NiO sensor in the Cl^–^ solutions ranged from 639 to 3284. In Na^+^ solutions, the ZnO sensor possessed detection sensitivities of 2.9–4.3. In the ZnO-based sensors, the high-number density NiO microparticles led to enhanced detection of Cl^–^ solutions, but not of Na^+^ and Ca^2+^ solutions. Compared to other SnO*_*x*_*-based sensors, the 4-SnO*_*x*_*/NiO sensor possessed the lowest and highest detection sensitivities (0.81 and 1.05) for 1 and 200 ppm of Cl^–^ solutions, respectively. Depending on the NiO microparticle number density, the detection sensitivities of the 4-SnO*_*x*_*/NiO sensors were high in the Na^+^ and Ca^2+^ solutions. For all of the sensors, the response times were mostly independent of the NiO microparticle number density, as shown in Figure 5d–f. For the CuO*_*x*_*-based sensors, the response times decreased and increased with increasing Cl^–^ and Na^+^ concentrations, respectively, and were independent of Ca^2+^ concentration. For the ZnO-based sensors, the response times were independent of the Cl^–^ concentration and increased with increasing Na^+^ and Ca^2+^ concentrations. For the SnO*_*x*_*-based sensors, the response times increased with increasing Cl^–^ concentration and were independent of the Na^+^ and Ca^2+^ concentrations. These results suggest that for certain ionic solutions, the optimal combinations of MOS materials and NiO microparticles effectively enhanced the detection sensitivities and response times of the sensors.

**Figure 5:**
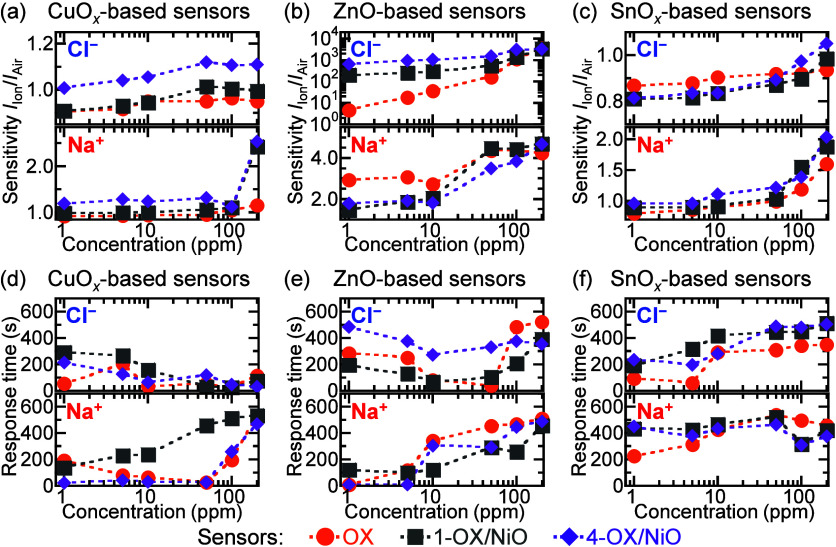
Detection sensitivities and response times of (a, d) CuO*_x_*-, (b, e) ZnO-, and (c, f) SnO_*x*_-based sensors, respectively, plotted as functions of Cl^–^ and Na^+^ concentrations.

The sensors’ operating temperature is one of the key factors in understanding the interactions between MOS materials and target molecules.^29–31^ Figure 6 shows the transient electrical responses of current ratios *I*_T_/*I*_Tair_ of 4-OX/NiO sensors in 100 ppm of Cl^–^ and Na^+^ solutions at operating temperatures ranging from 10 to 50 °C, where *I*_T_ and *I*_Tair_ are the currents measured in the solution and air, respectively, and depend on the operating temperature. To prevent the evaporation of the ionic solutions during high-temperature sensor measurements, the sensors operating at 10, 20, 30, 40, and 50 °C were exposed to the ionic solutions for 60, 50, 40, 30, and 20 min, respectively. The 4-CuO*_*x*_*/NiO sensors’ currents in Cl^–^ and Na^+^ solutions increased at 10 °C and decreased then increased at 50 °C. The current ratios of the 4-CuO*_*x*_*/NiO sensor in ionic solutions were higher at lower operating temperatures than at higher operating temperatures. However, over time, the current change rates of the 4-CuO*_*x*_*/NiO sensor in ionic solutions were higher at higher operating temperatures than at lower operating temperatures. For the 4-ZnO/NiO sensor in the ionic solutions, the measured currents increased and then saturated, independent of the sensors’ operating temperature. At all of the operating temperatures, the 4-ZnO/NiO sensor measured currents of approximately 1 mA and 10 μA in Cl^–^ and Na^+^ solutions, respectively. At all the operating temperatures, the 4-SnO*_*x*_*/NiO sensors’ currents in the ionic solutions increased. With an increase in operating temperature, the current changes of the SnO*_*x*_*-based sensors accelerated. The transient electrical responses of CuO*_*x*_*-, ZnO-, and SnO*_*x*_*-based sensors operating at different temperatures in 100 ppm ionic solutions are shown in Figures S4–S6, respectively. The transient electrical responses of all of the sensors in the Ca^2+^ and Na^+^ solutions showed the same trend. NiO microparticles affected the operating temperature dependence of the sensing performance of the MOS-based sensors in the ionic solutions.

**Figure 6:**
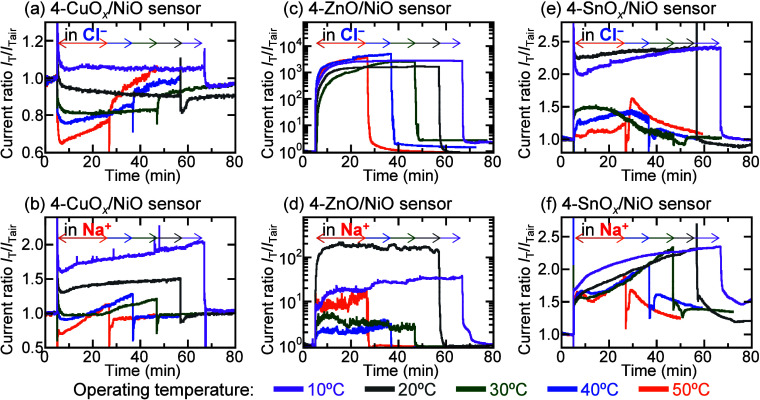
Transient electrical responses of the (a, b) 4-CuO_*x*_/NiO, (c, d) 4-ZnO/NiO, and (e, f) 4-SnO_*x*_/NiO sensors operating at different temperatures in the 100 ppm of Cl^–^ and Na^+^ solutions, respectively.

Arrhenius plots were constructed to estimate the sensors’ activation energies, the initial energy required for the reaction and adsorption of molecules on the MOS surface,^32,33^ from the effect of the operating temperature on the ion detection sensitivity. Figure 7 shows the Arrhenius plots of the resistance change (ln­(*R*_Ion_/*R*_Air_)), 20 min after dropping 100 ppm of Cl^–^ and Na^+^ ionic solutions, as functions of the reciprocal of the absolute operating temperature (1/*T*) for the OX and 4-OX/NiO sensors, where *R*_Ion_ and *R*_Air_ are the sensors’ resistances measured in ionic solution and air, respectively. Arrhenius plots for all sensors are shown in Figures S4–S6. For all of the sensors, the resistance changes were approximately negatively proportional to the reciprocal of the operating temperature. For all of the 4-OX/NiO sensors, the slopes of the Arrhenius plots were gentler than those for the OX and 1-OX/NiO sensors. The activation energies of all the sensors operating in all the 100 ppm ionic solutions were estimated from the slopes of the Arrhenius plots in Figure 7. The sensors’ activation energy (*E*_a_) is given by^34–36^

$$\frac{R_{\mathrm{Ion}}}{R_{\mathrm{Air}}}=R_{0}\exp\left(-\frac{E_{a}}{k_{B}T}\right)$$ 1

where *R*_0_ is the pre-exponential factor, *k*_B_ is the Boltzmann constant, and *T* is the absolute temperature. Table 1 shows the activation energies of all sensors operating in each 100 ppm ionic solution. The activation energies of the CuO*_*x*_*-, ZnO-, and SnO*_*x*_*-based sensors operating in the Cl^–^ solution decreased from 111.4 to 64.5, 330.2 to 215.1, and 160.1 to 137.3 meV, respectively, with increasing ion concentration, which are reasonable compared to those of previously reported MOS materials.^37–39^ The activation energies of the NiO microparticle-bearing sensors were lower than those of the NiO microparticle-free sensors. Because the activation energies of all the sensors operating in the Cl^–^, Na^+^, and Ca^2+^ solutions showed a similar trend, the NiO microparticles led to a decrease in the activation energies of the MOS-based sensors operating in the ionic solutions and, therefore, an enhanced reaction between the MOS nanostructures and ions.

**Figure 7:**
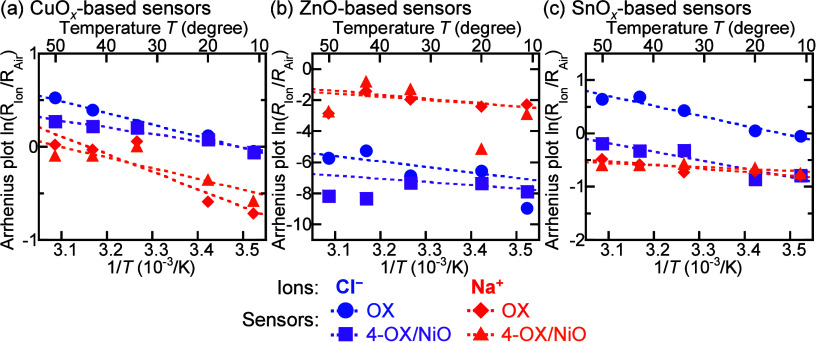
Arrhenius plots of the (a) CuO_*x*_-, (b) ZnO-, and (c) SnO_*x*_-based sensors operating in the 100 ppm of Cl^–^ and Na^+^ solutions.

**Table 1:** Activation Energies of MOS-Based Sensors Operating in Each 100 ppm of Ionic Solution

	activation energy (meV)								
	CuO*_*x*_*-based sensors			ZnO-based sensors			SnO*_*x*_*-based sensors		
ion (100 ppm)	CuO*_*x*_*	1-CuO*_*x*_*/NiO	4-CuO*_*x*_*/NiO	ZnO	1-ZnO/NiO	4-ZnO/NiO	SnO*_*x*_*	1-SnO*_*x*_*/NiO	4-SnO*_*x*_*/NiO
Cl^–^	111.4	93.2	64.5	330.2	212.5	215.1	160.1	140.2	137.3
Na^+^	162.2	102.9	98.9	223.5	182.0	148.5	59.3	32.5	29.7
Ca^2+^	32.5	31.5	24.8	237.6	187.1	94.6	57.0	50.8	39.4

### Sensors’ Mechanism for Detecting Ions in Solutions

MOS-based sensors detect target molecules primarily based on changes in the sensors’ electrical properties.^17,40^ The detection mechanism of MOS-based sensors operating in ionic solutions can be explained by the changes in the number of electrons on the sensors’ surface caused by reactions between the MOS and ionic solution. CuO, Cu_2_O, SnO, and NiO are *p*-type semiconductors, where holes are the majority carriers,^41^ while ZnO and SnO_2_ are *n*-type semiconductors, where electrons are the majority carriers.^42^ The currents of the CuO*_*x*_* and SnO*_*x*_* sensors and that of the ZnO sensor operating in DI water were lower and higher, respectively, than the currents of those sensors operating in air (Figure S7). In addition, the NiO microparticles led to a decrease in the current of the sensors operating in DI water. Thus, the MOS reacted with water molecules (given by reaction 1i, where the subscripts *ad* and *O* mean the species adsorbed on the MOS’s surface and occupying lattice oxygen sites in the MOS, respectively; O_O_ and V_O_ are lattice oxygen atoms and oxygen vacancies in the MOS, respectively; and e^–^ is an electron), increasing the number of electrons on the MOS’s surface.^43,44^

$$\mathrm{Metal}+O_{O}+H_{2}O↔\mathrm{Metal}{\left(\mathrm{OH}\right)}_{\mathrm{ad}}+V_{O}+e^{-}$$ i

$$O_{O}↔V_{O}+O^{2-}$$ ii

The MOS-based sensors’ currents increased with increasing ion concentration in the ionic solutions (Figure 4). This is because the reactions between the MOS and ionic solutions led to a decrease and an increase in the number of electrons on the CuO*_*x*_* and SnO surfaces and on the ZnO and SnO_2_ surfaces, respectively. These reactions occurred between the MOS’s bulk^45,46^ and surface^47,48^ and ions in the solutions. Figure 8 shows an example of these bulk and surface reactions. On the bulk and surface of the MOS, the change in the number of electrons caused by the ion transfer between them and the ionic solutions would be given by the following reactions.

$${\mathrm{Cl}}^{-}+V_{O}+e^{-}↔{\mathrm{Cl}}_{O}^{-}$$ iii

$${\mathrm{Cl}}^{-}+O_{O}↔{\mathrm{Cl}}_{O}^{-}+O_{2}+e^{-}$$ iv

$$\mathrm{Metal}{\left(\mathrm{OH}\right)}_{\mathrm{ad}}+{\mathrm{Na}}^{+}↔\mathrm{Metal}{\left(\mathrm{ONa}\right)}_{\mathrm{ad}}+H^{+}$$ v

$$\mathrm{Metal}{\left(\mathrm{OH}\right)}_{\mathrm{ad}}+{\mathrm{Cl}}^{-}+H^{+}↔\mathrm{Metal}{\left({\mathrm{OH}}_{2}\mathrm{Cl}\right)}_{\mathrm{ad}}$$ vi

$$H^{+}+e^{-}↔H_{\mathrm{ad}}$$ vii

When the MOS’s surface was exposed to the ionic solutions, the number of electrons on the MOS’s surface changed, mainly because the oxygen vacancies in the *p*-type CuO*_*x*_* (reaction [Disp-formula eq1iii]) and the lattice oxygen atoms in the *n*-type ZnO (reaction [Disp-formula eq1iv]) were replaced by ions. In the SnO*_*x*_*-based sensors, which possessed both *p*- and *n*-type semiconductors, both reactions [Disp-formula eq1iii] and [Disp-formula eq1iv] occurred. In addition, on the MOS’s surface, the adsorption of cations (reaction 1v) and anions (reaction [Disp-formula eq1vi]) decreased and increased the number of electrons because of the adsorption/desorption of protons,^49^ respectively. The larger ionic valence and ionization tendency may lead to the activation of these reactions, such as the higher detection sensitivity of the CuO*_*x*_* sensor to Ca^2+^ than Na^+^, as shown in Figures 5 and S3. The lower operating temperatures led to higher currents in MOS-based sensors (Figures S4–S6). At lower operating temperatures, bulk reactions [Disp-formula eq1iii] and [Disp-formula eq1iv] may predominate, while at higher operating temperatures, surface reactions 1v and [Disp-formula eq1vi] may predominate due to the activation of the formation of hydroxyl groups and oxygen ions on the MOS surface.^50,51^ These reactions, which depend on the MOS materials, ion species, and operating temperatures, enabled the MOS-based sensor to detect ions in solutions. Furthermore, the EDX spectra of all sensors’ surfaces after dropping and then removing the 100 pm ionic solutions (Figure S8) were the same as before dropping them (Figures 3 and S2). In addition, all sensors’ currents changed and then recovered after dropping the ionic solution on and then removing it from the sensors’ surfaces, respectively (Figures 4 and S3). Therefore, it is suggested that the bulk and surface states of the MOS, changed by the reactions with the ionic solutions, are returned to their original states after exposure to air.

**Figure 8:**
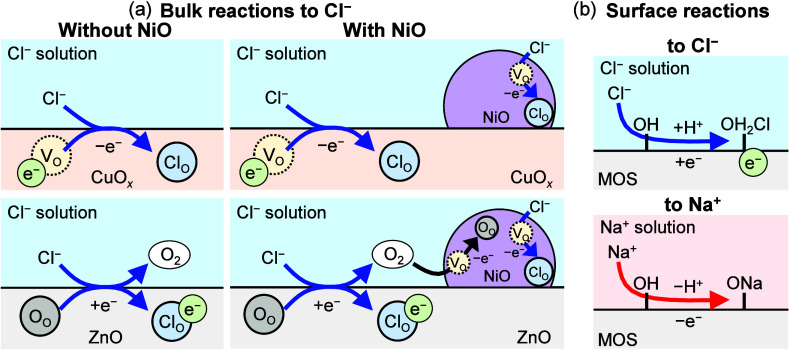
Illustrations of (a) bulk reactions of CuO_*x*_, ZnO, and NiO with Cl^–^ and (b) surface reactions of the MOS material with Cl^–^ and Na^+^.

The *p*–*p* and *p*–*n* heterojunctions are important factors affecting the detection sensitivities of MOS-based sensors.^52–54^ NiO forms *p*–*p* and *p*–*n* heterojunctions with CuO*_*x*_* and ZnO, respectively. The SnO*_*x*_*/NiO sensor may possess both *p*–*p* and *p*–*n* heterojunctions. The presence of these *p*–*p* and *p*–*n* heterojunctions is discussed in Figure S9. Because the CuO*_*x*_* sensor and the NiO microparticles in the CuO*_*x*_*/NiO sensor reacted with the ionic solutions in the same manner, the CuO*_*x*_*/NiO *p*–*p* heterojunctions led to enhanced detection sensitivity in ionic solutions. On the other hand, in the ZnO/NiO sensor, the reactions between the NiO and ionic solutions depend on the ion species. In the Cl^–^ solution, NiO’s oxygen vacancy is reduced by oxygen atoms transferred from ZnO through reaction [Disp-formula eq1iv]. In contrast, in the Na^+^ and Ca^2+^ solutions, the reactions between NiO and the ionic solutions (reaction [Disp-formula eq1iii]) are promoted by removing the electrons generated by reaction [Disp-formula eq1iv] in ZnO. Therefore, the ZnO/NiO sensor’s currents in low-concentration Cl^–^ solution and Na^+^ and Ca^2+^ solutions were higher and lower, respectively, than the ZnO sensor’s currents in the same solutions. In high-concentration ionic solutions, the NiO microparticles did not affect the detection sensitivity of the ZnO-based sensors because NiO’s oxygen vacancies were filled. The *p*–*p* and *p*–*n* heterojunctions formed between the MOS and NiO, which lead to a decrease in the sensor’s activation energy and substantially modulate the current, contribute to the enhanced detection sensitivity of the MOS-based sensors to ions in solutions.

## Conclusions

We fabricated *p*–*p* and *p*–*n* heterojunctions between MOS nanostructures and NiO microparticles on metallic foils and evaluated their ion sensing performance in Cl^–^, Na^+^, and Ca^2+^ solutions. MOS/NiO heterojunctions were fabricated by the formation of CuO and Cu_2_O nanowires, ZnO nanowires, or SnO_2_ and SnO nanoparticles by heating metallic foils, followed by the deposition of NiO microparticles using Ni­(OH)_2_-containing colloidal solutions. The NiO microparticles enhanced the detection sensitivities of the CuO*_*x*_*- and SnO*_*x*_*-based sensors operating in all of the ionic solutions. In contrast, the ZnO/NiO sensor fabricated using 4 mg of Ni­(OH)_2_ possessed the highest ion detection sensitivity (639) in the 1 ppm Cl^–^ solution, and the ZnO sensor (2.9 and 1.6) in the Na^+^ and Ca^2+^ solutions, respectively. The NiO microparticle-bearing MOS-based sensors possessed lower activation energies than the NiO microparticle-free MOS-based sensors. These results suggest that for the MOS-based sensors operating in the ionic solutions, the *p*–*p* and *p*–*n* heterojunctions led to enhanced ion detection sensitivities by promoting bulk and surface reactions between the MOS and ions. The information in this study will enable the design of advanced high-performance MOS-based chemiresistive ion sensors.

## Supplementary Material



## Notes

Scanning electron microscopy and optical microscopy images of heated metallic foil surfaces, energy-dispersive X-ray spectroscopy spectra and X-ray diffraction patterns, sensing performance of ion sensors operating in Ca^2+^ solutions, transient electrical responses and Arrhenius plots of CuO*_*x*_*-based sensors operating at different temperatures, transient electrical responses and Arrhenius plots of ZnO-based sensors operating at different temperatures, transient electrical responses and Arrhenius plots of SnO*_*x*_*-based sensors operating at different temperatures, transient electrical responses of sensors operating in deionized water, energy-dispersive X-ray spectroscopy spectra after dropping and then removing the ionic solutions, and impedance spectroscopy data of all sensors (PDF)
