# Supplementary material for: Metal-Oxide-Semiconductor Nanostructure/NiO Microparticle Heterojunctions Formed on Metallic Foils for Sensitive Chemiresistive Ion Sensors
Source: ACS Omega. 2025 Jun 20;10(25):27323–31. doi: 10.1021/acsomega.5c02828 (PMC12224104; doi:10.1021/acsomega.5c02828)
Supplement: Supplementary file 1 [file ao5c02828_si_001.pdf]

Supporting Information

Metal-Oxide-Semiconductor Nanostructure/NiO Micro-particle Heterojunctions Formed on Metallic Foils for Sensitive Chemiresistive Ion Sensors

Yoshinari Kimura* and Hironori Tohmyoh*

Department of Finemechanics, Graduate School of Engineering, Tohoku University, 6-6-01 Aoba, Aramaki, Aoba-ku, Sendai 980-8579, Japan

Corresponding authors: yoshinari.kimura.a5@tohoku.ac.jp (Y. Kimura),

hironori.tohmyoh.e6@tohoku.ac.jp (H. Tohmyoh)

Contents

Figure S1. Scanning electron microscopy and optical microscopy images of heated metallic foil surfaces.

Figure S2. Energy-dispersive X-ray spectroscopy spectra and X-ray diffraction patterns.

Figure S3. Sensing performance of ion sensors operating in Ca^{2+} solutions.

Figure S4. Transient electrical responses and Arrhenius plots of CuO_x-based sensors operating at different temperatures.

Figure S5. Transient electrical responses and Arrhenius plots of ZnO-based sensors operating at different temperatures.

Figure S6. Transient electrical responses and Arrhenius plots of SnO_x-based sensors operating at different temperatures.

Figure S7. Transient electrical responses of sensors operating in deionized water.

Figure S8. Energy-dispersive X-ray spectroscopy spectra after dropping and then removing the ionic solutions.

Figure S9. Impedance spectroscopy data of all sensors.

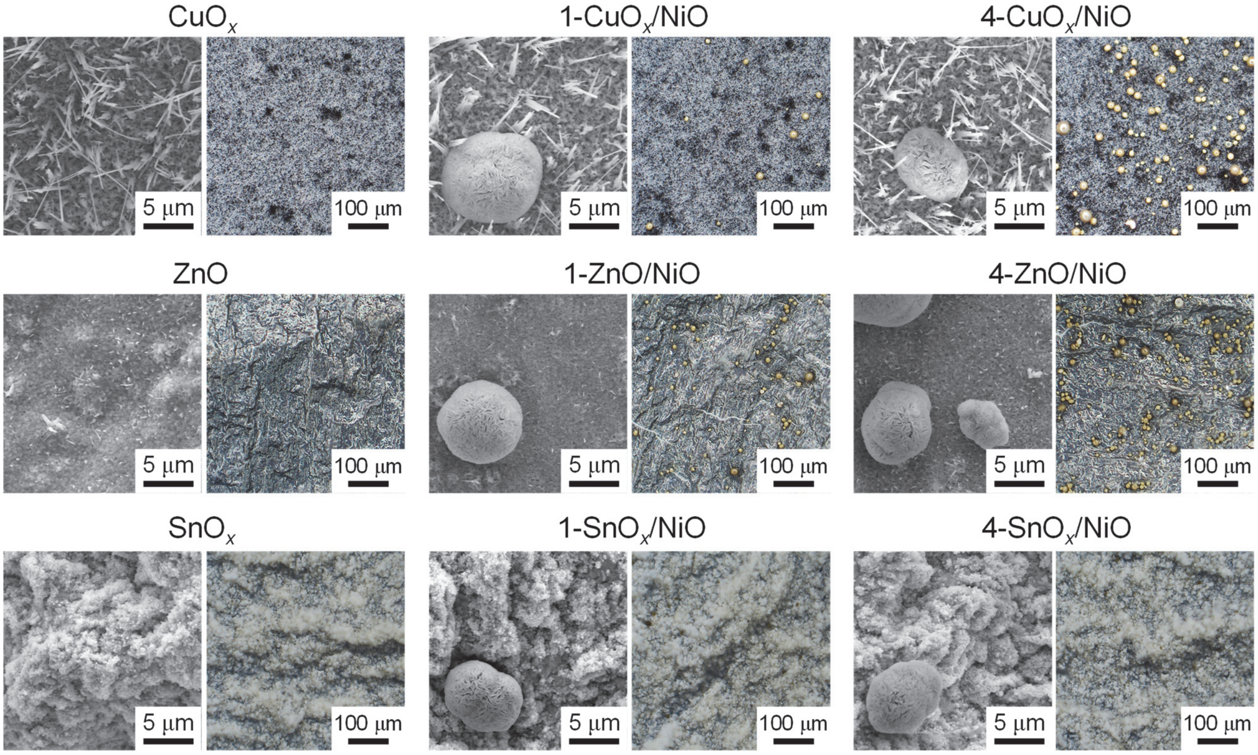

Figure S1. Scanning electron microscopy and optical microscopy images of nanostructures and microparticles on the surfaces of the heated metallic foils.

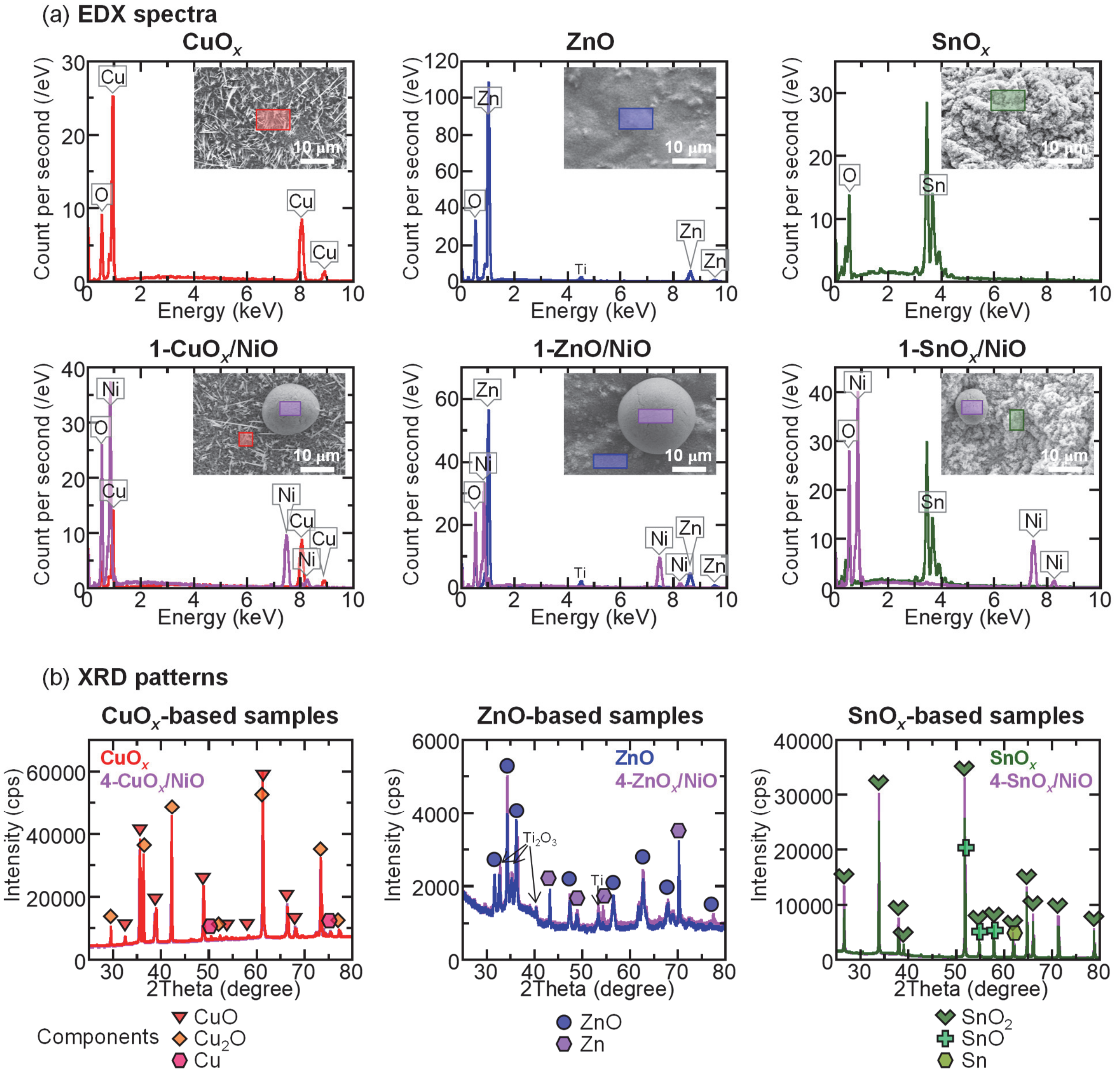

Figure S2. (a) Energy-dispersive X-ray spectroscopy (EDX) spectra and (b) X-ray diffraction (XRD) patterns of the heated metallic foils.

In the 4-CuO$_x$/NiO and 4-SnO$_x$/NiO samples, NiO peaks could not be identified because the NiO peak intensities are much lower than those related to Cu and Sn.

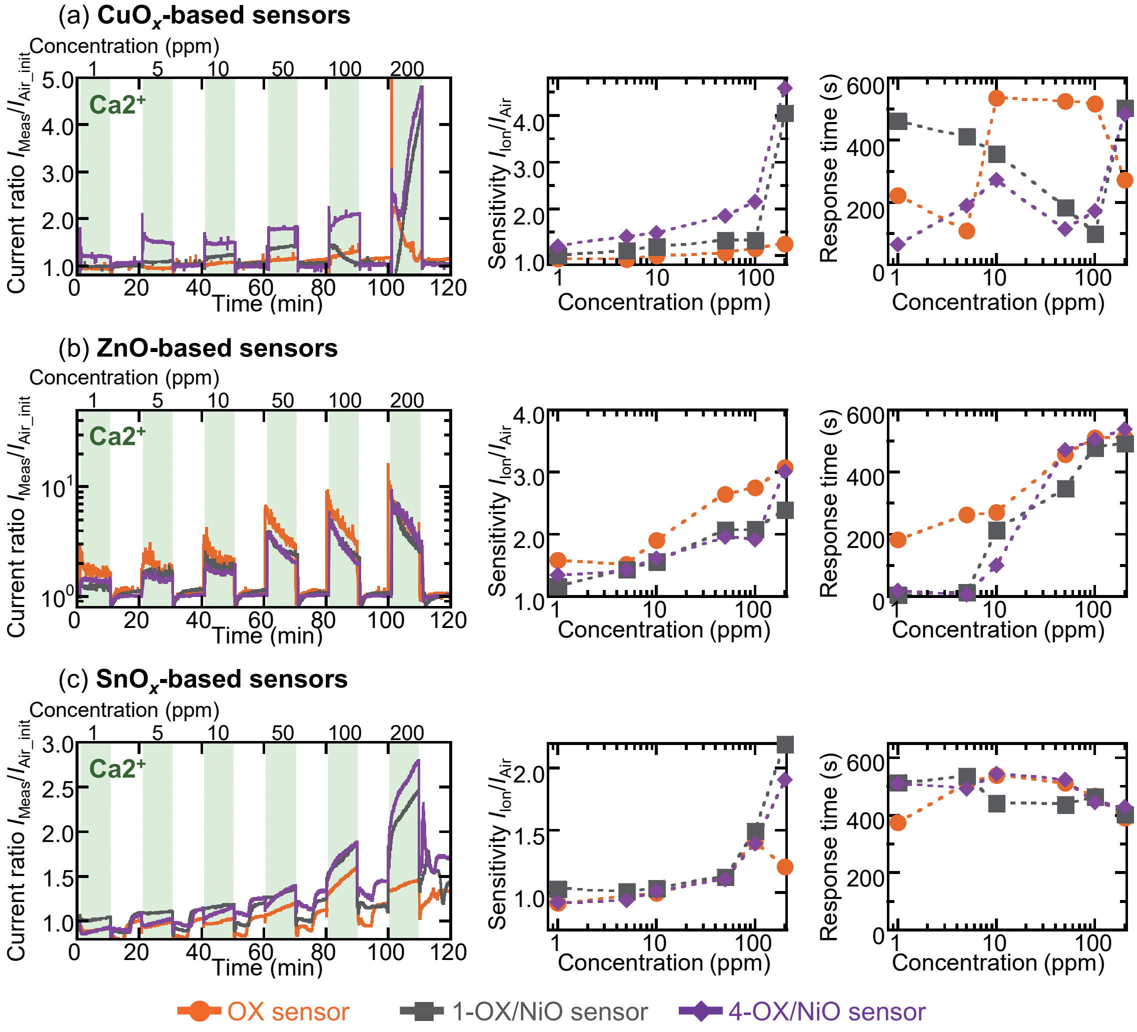

Figure S3. Transient electrical responses, detection sensitivities, and response times of all the sensors operating in the Ca^{2+} solution.

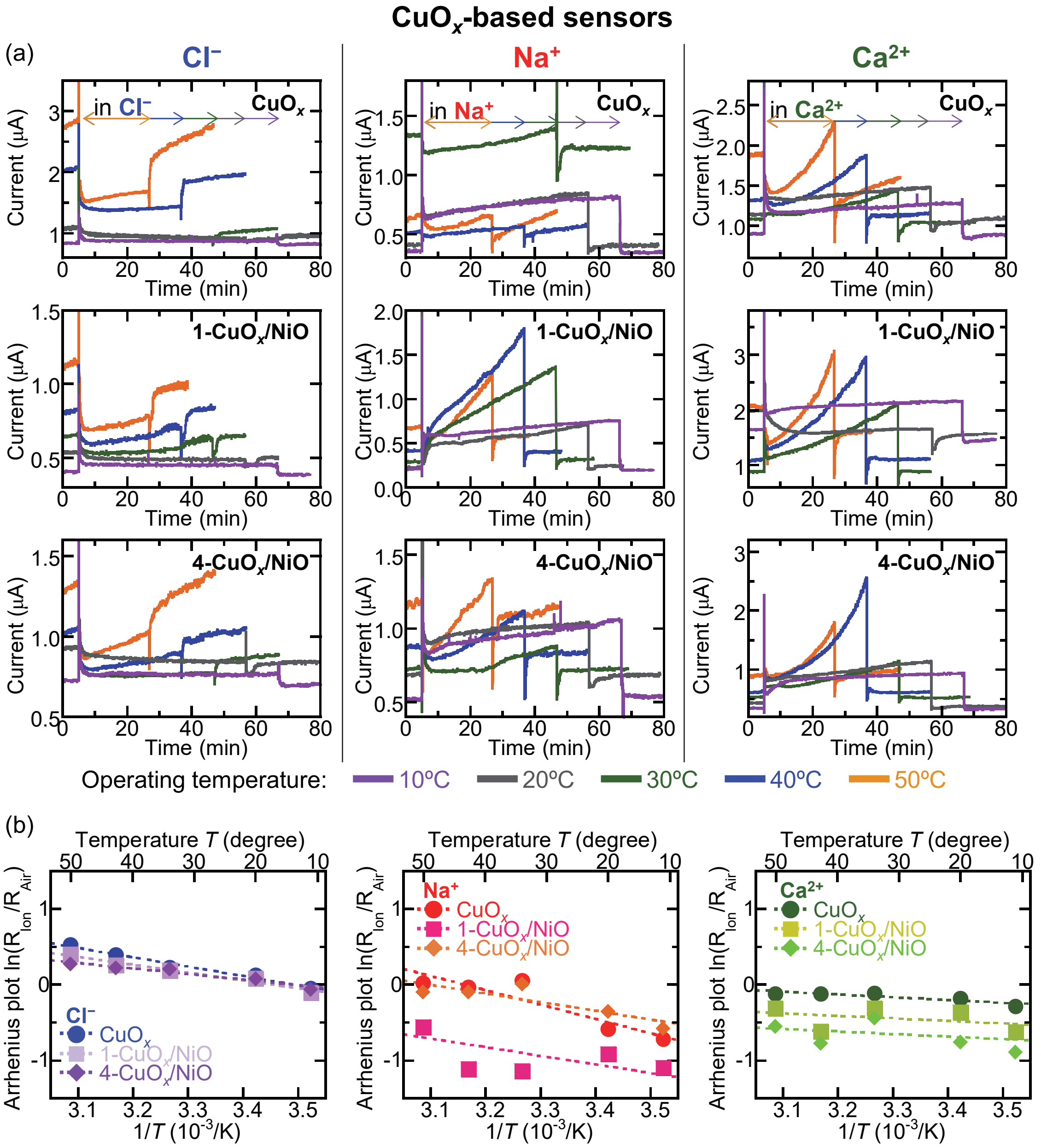

Figure S4. (a) Transient current responses and (b) Arrhenius plots of CuO_x-based sensors operating at different temperatures in the 100 ppm ionic solutions.

Different CuO_x sensors were used for the measurements in the Na^+ solution at 30°C and the other operating temperatures.

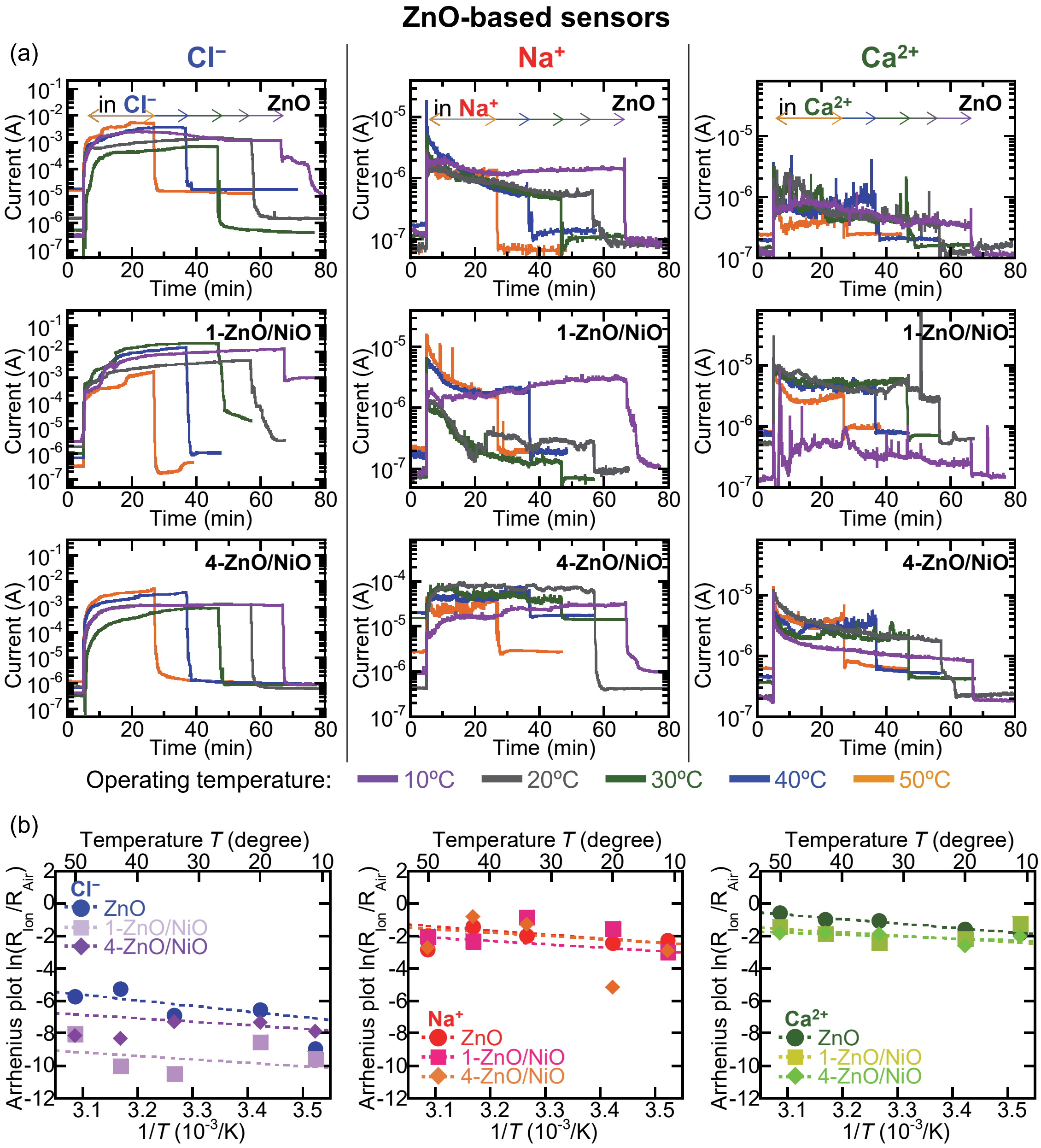

Figure S5. (a) Transient current responses and (b) Arrhenius plots of ZnO-based sensors operating at different temperatures in the 100 ppm ionic solutions.

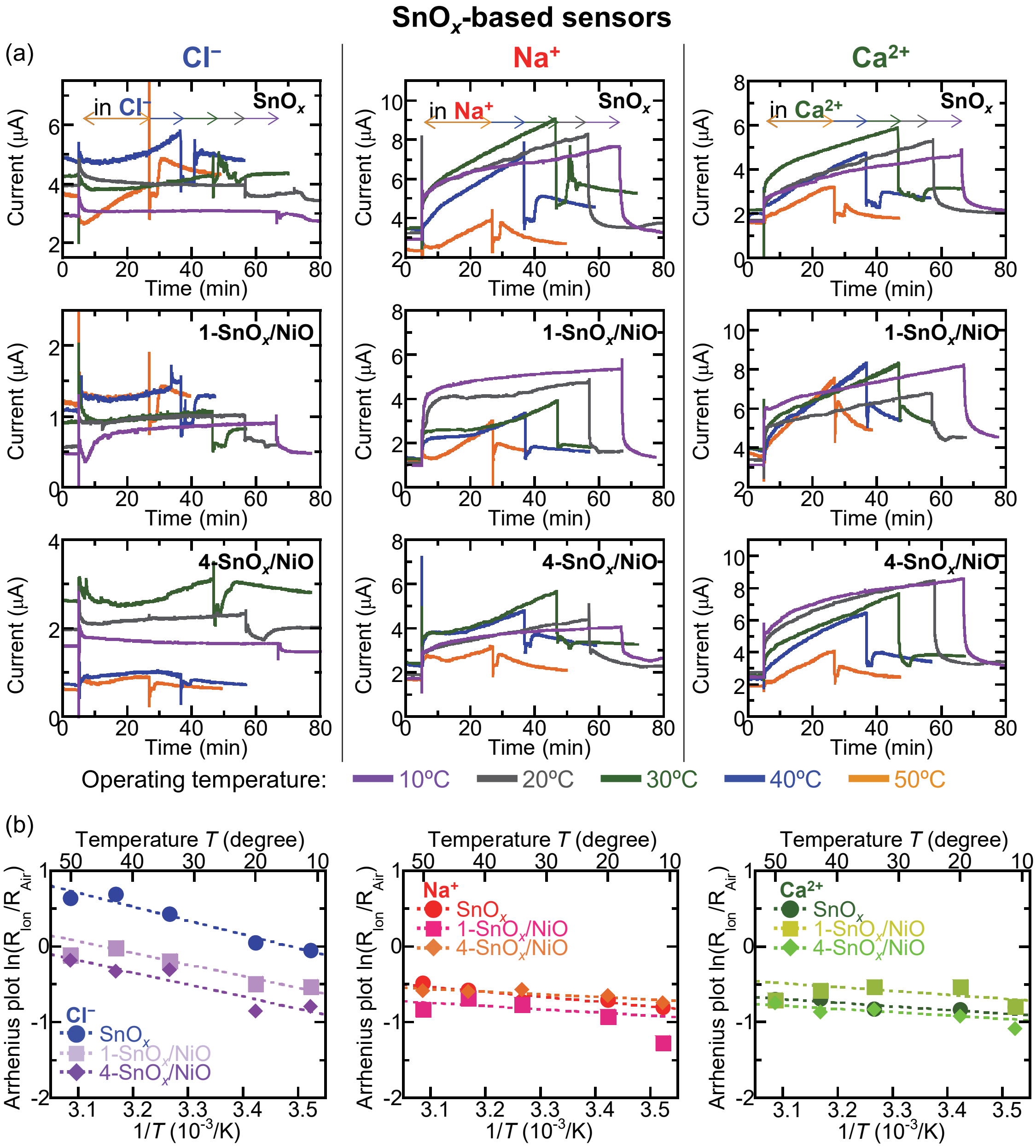

Figure S6. (a) Transient current responses and (b) Arrhenius plots of SnO_x-based sensors operating at different temperatures in the 100 ppm ionic solutions.

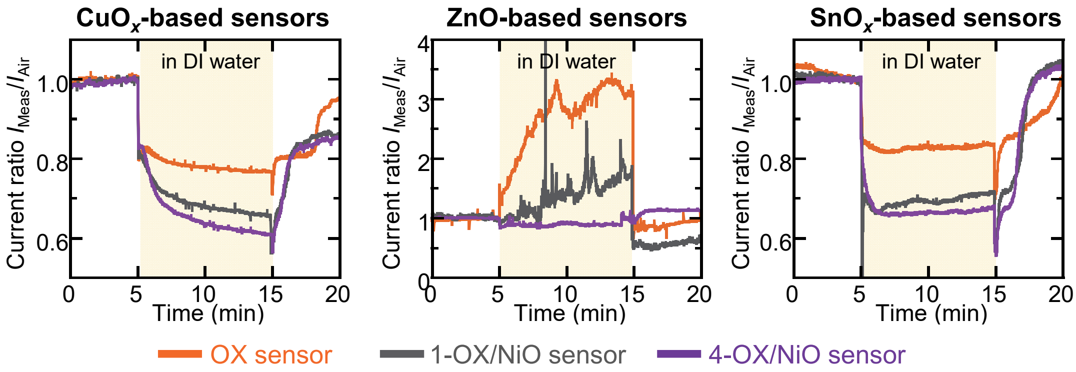

Figure S7. Transient electrical responses of all the sensors operating at 30°C in deionized water.

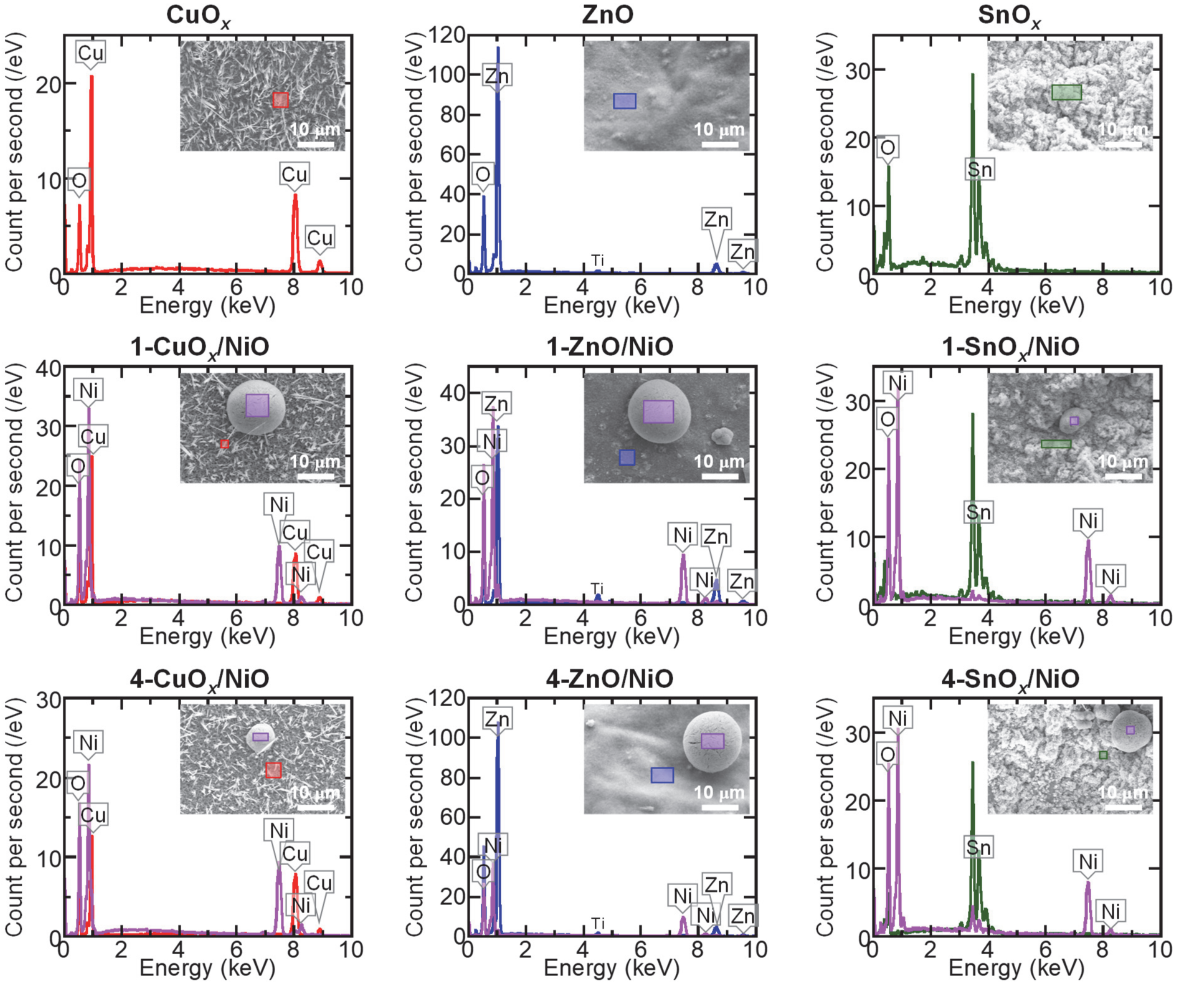

Figure S8. Energy-dispersive X-ray spectroscopy spectra of all sensors' surfaces after dropping and then removing the 100 ppm all ionic solutions.

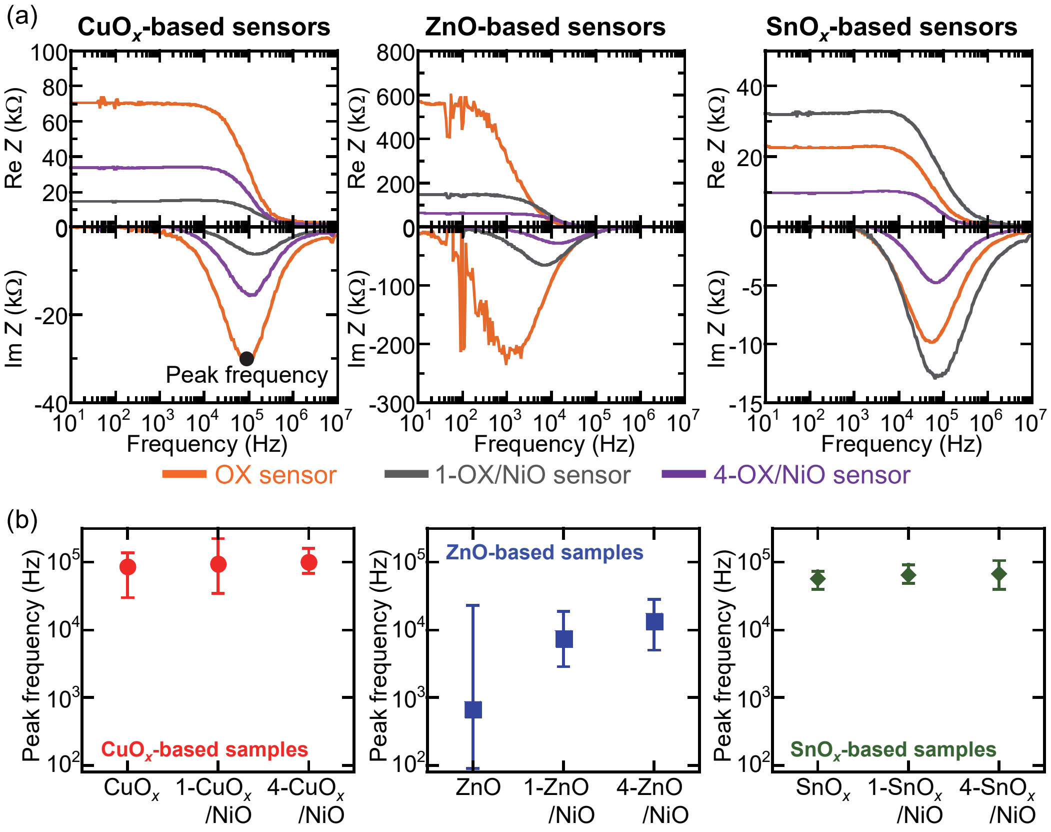

Figure S9. (a) Real (Re *Z*) and imaginary (Im *Z*) part of impedances of all samples as functions of frequency. (b) Peak frequency observed in the imaginary part of the impedance.

The impedance spectroscopy was measured in air using an impedance analyzer (Analogue Discovery 2, Digilent) at frequencies from 10 Hz to 10 MHz, an amplitude voltage of 0.1 V, and a bias voltage of 0 V. The Im *Z* characteristics of all samples showed one large peak. This behavior of Im *Z* implies that there is a capacitance component in the sample.[S1] The peak frequencies of Im *Z*, which correlate with the capacitance values, increased with increasing NiO microparticle number density. In particular, the peak frequencies of the *n*-type ZnO samples were very much affected by *p*-type NiO. Thus, it is suggested that there is a capacitance component between the MOS and NiO microparticles. From these results, NiO would forms *p*–*p* and *p*–*n* heterojunctions with the MOS because a depletion layer is formed at their heterojunction interface.[S2]

REFERENCES

(S1) Coskun, M.; Turut, A.; Ejderha, K. Frequency and Temperature-Dependent Electrical Characteristics of Ni/n-GaP/Al Schottky Barrier Diodes. *J. Mater. Sci.: Mater. Electron.* **2023**, 34, 1846.

(S2) Mathew, M.; Shinde, P. V.; Samal, R.; Rout, C. S. A Review on Mechanisms and Recent Developments in p-n Heterojunctions of 2D Materials for Gas Sensing Applications. *J. Mater. Sci.* **2021**, 56, 9575–9604.
